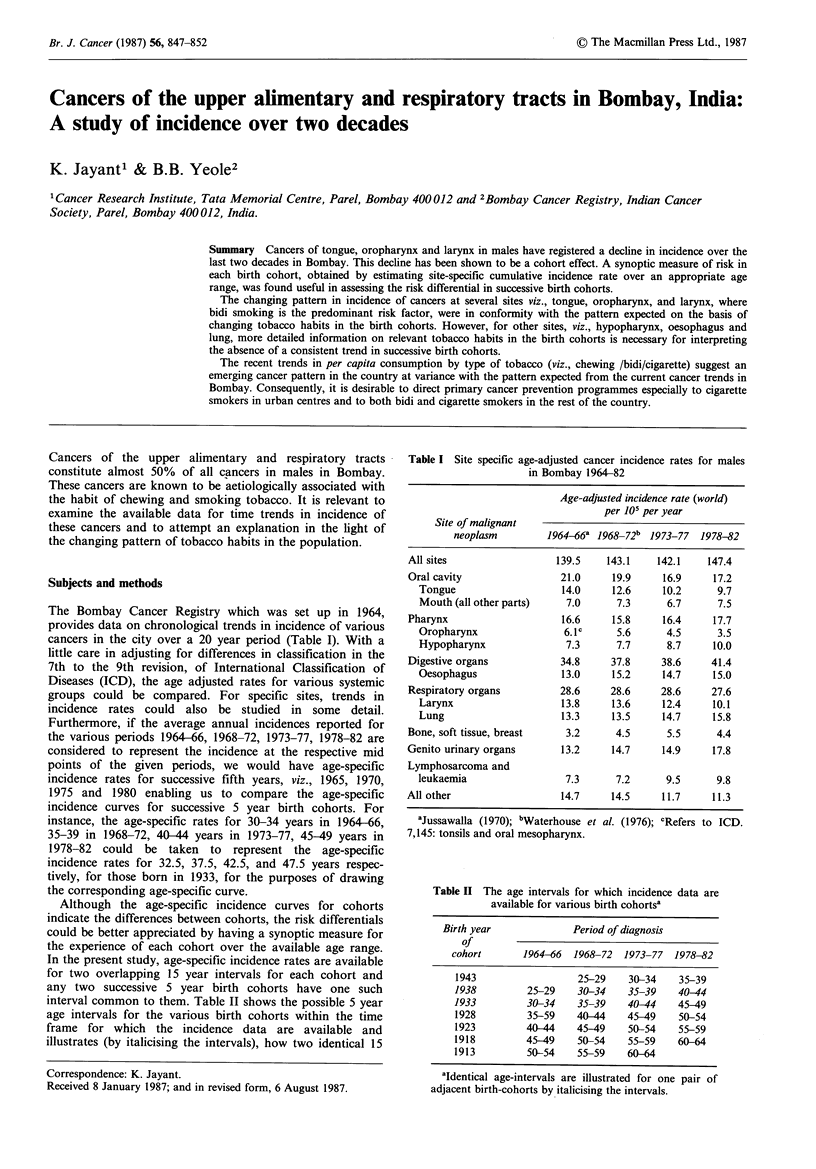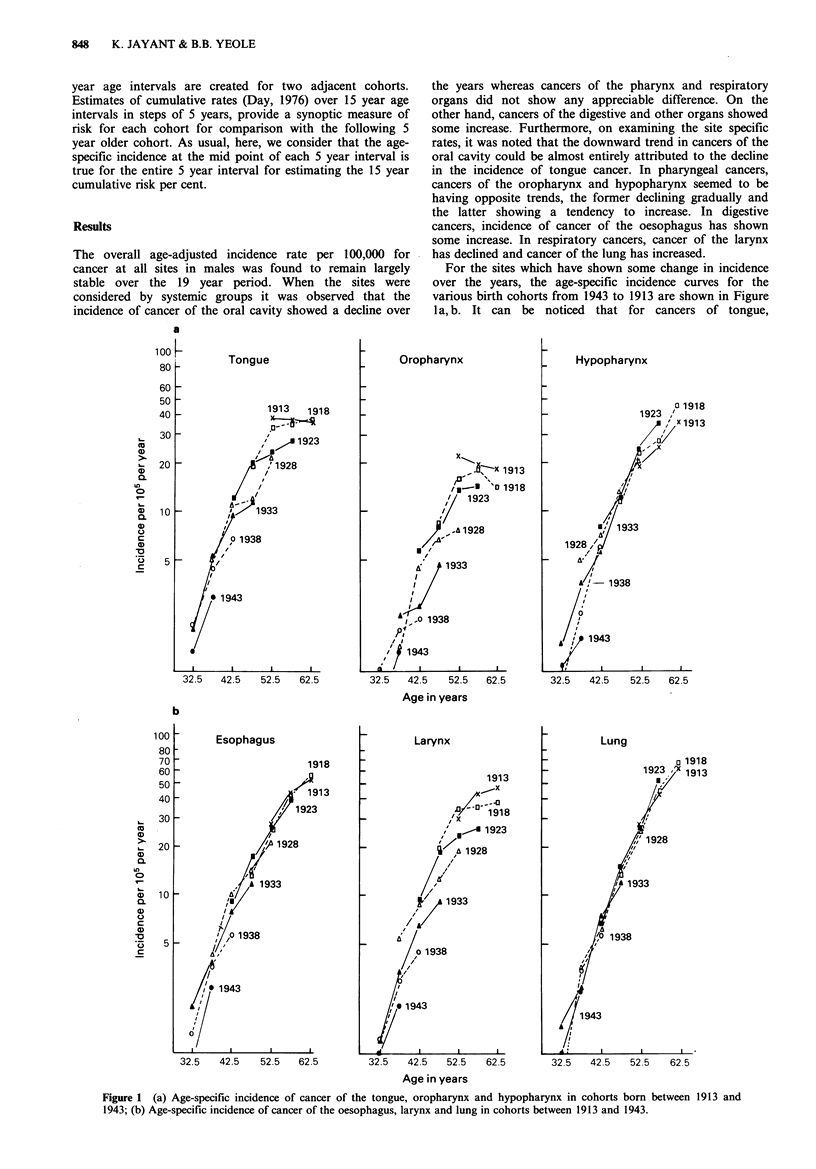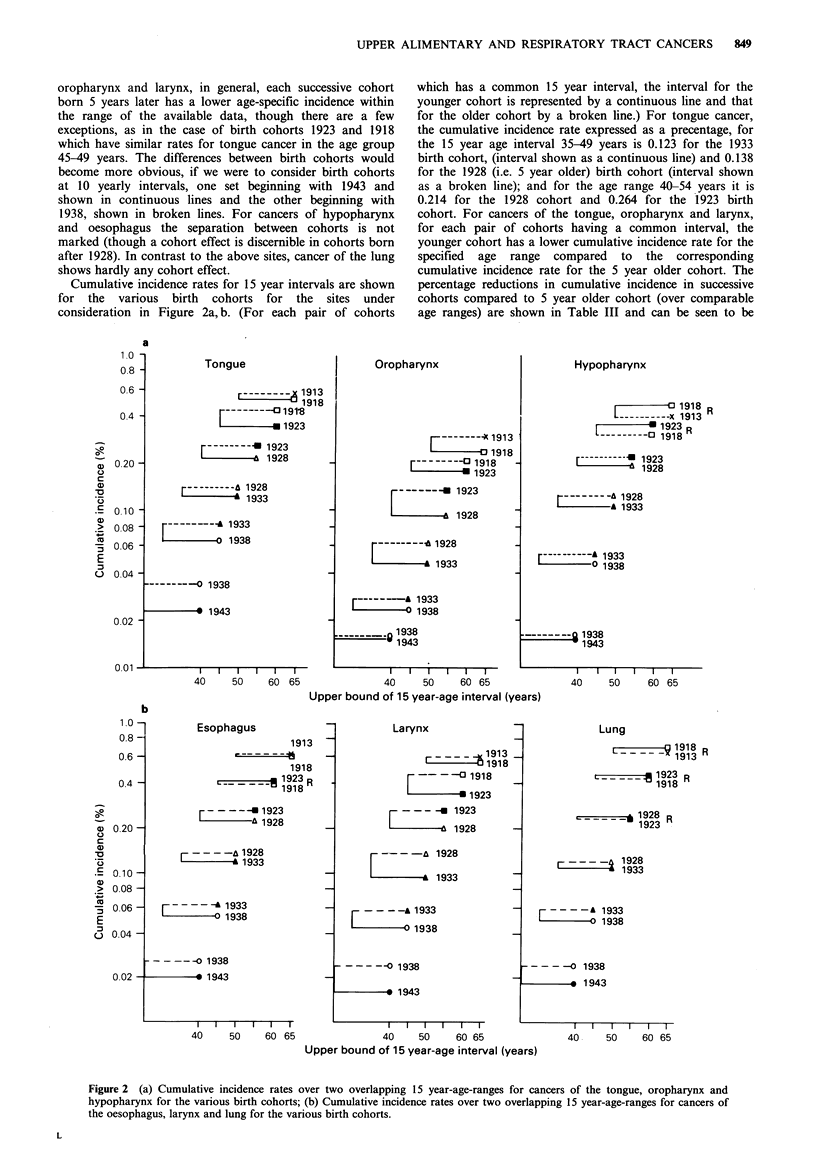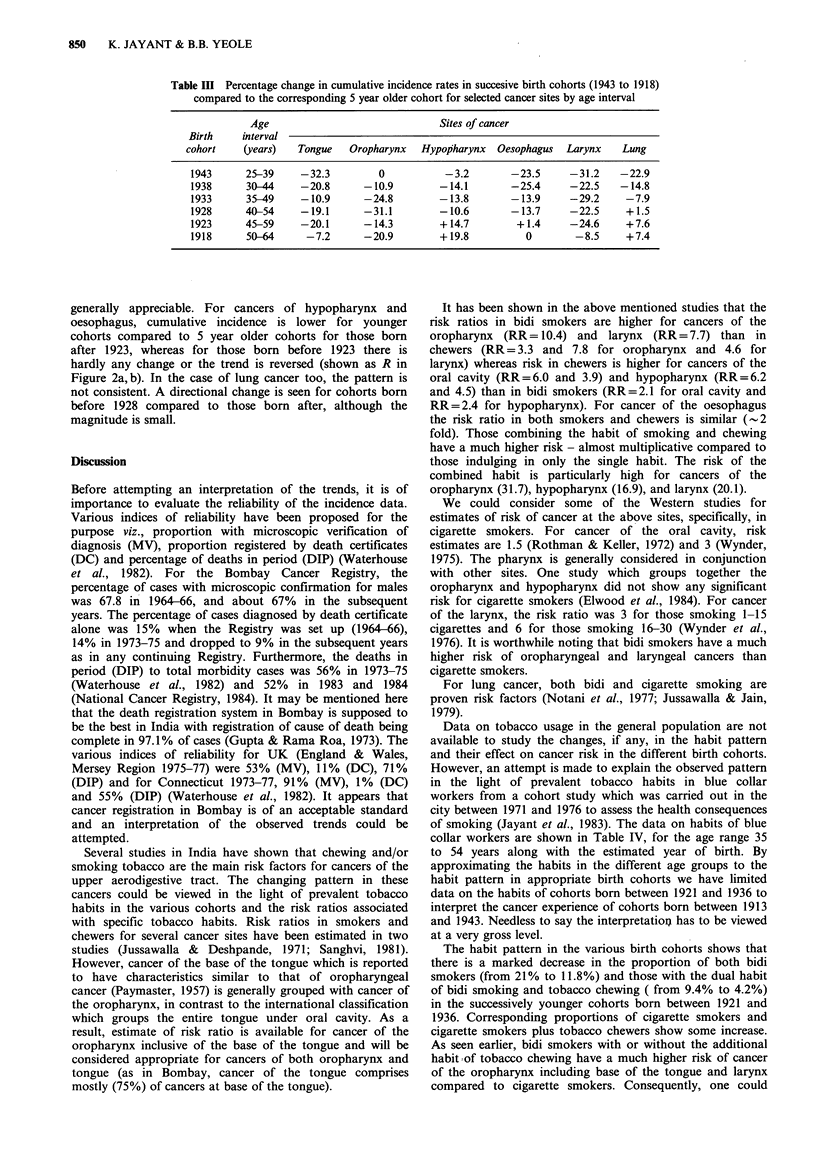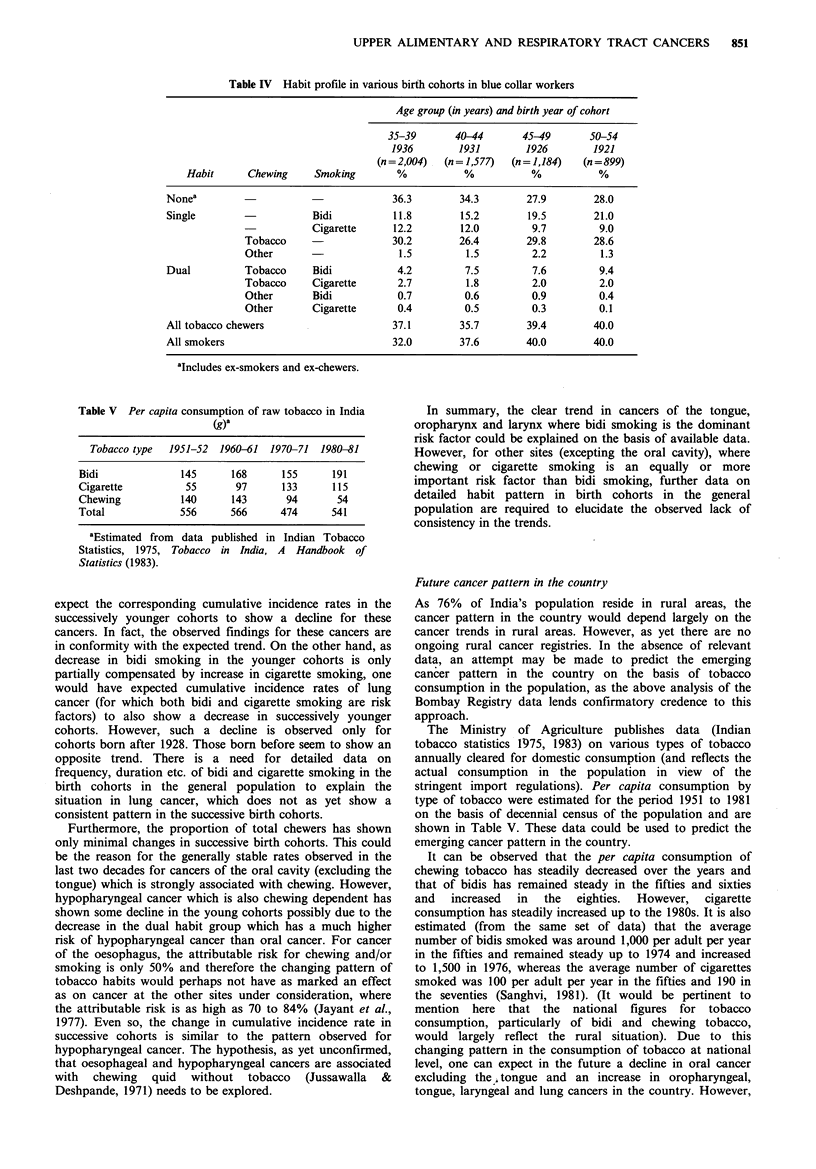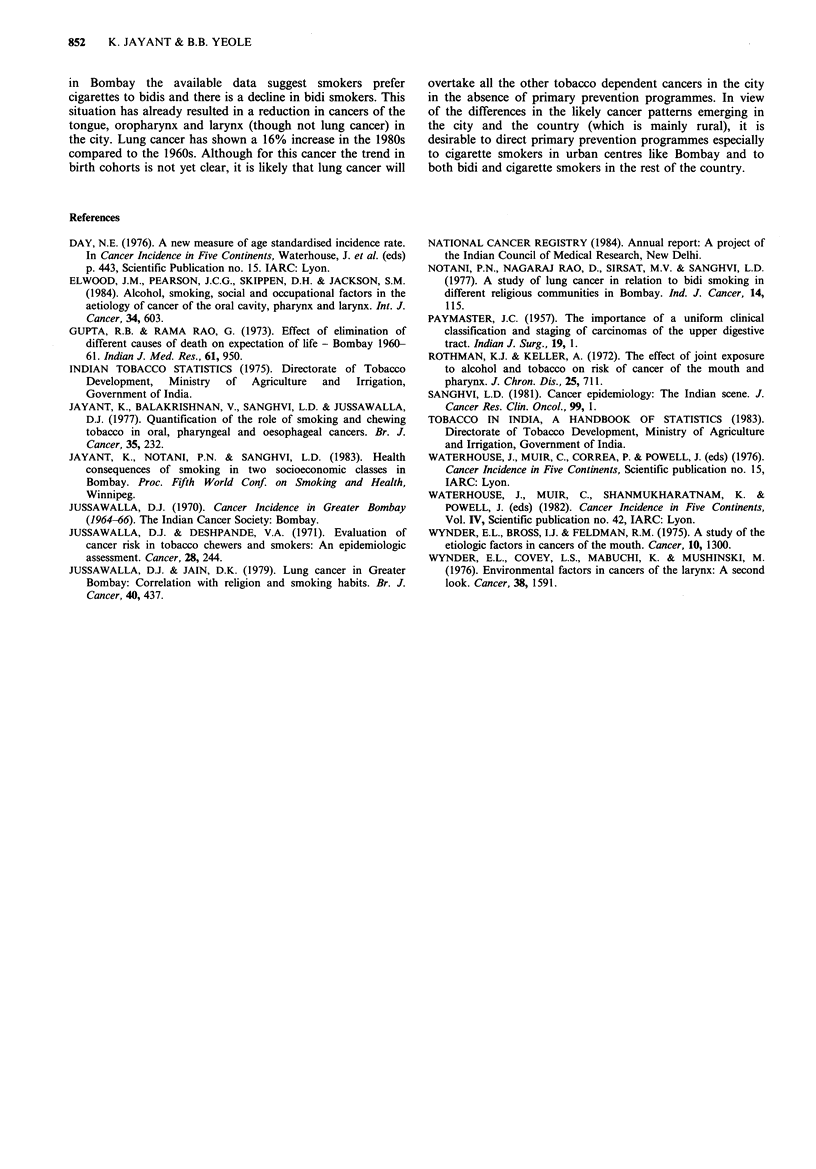# Cancers of the upper alimentary and respiratory tracts in Bombay, India: a study of incidence over two decades.

**DOI:** 10.1038/bjc.1987.304

**Published:** 1987-12

**Authors:** K. Jayant, B. B. Yeole

**Affiliations:** Cancer Research Institute, Tata Memorial Centre, Parel, Bombay, India.

## Abstract

Cancers of tongue, oropharynx and larynx in males have registered a decline in incidence over the last two decades in Bombay. This decline has been shown to be a cohort effect. A synoptic measure of risk in each birth cohort, obtained by estimating site-specific cumulative incidence rate over an appropriate age range, was found useful in assessing the risk differential in successive birth cohorts. The changing pattern in incidence of cancers at several sites viz., tongue, oropharynx, and larynx, where bidi smoking is the predominant risk factor, were in conformity with the pattern expected on the basis of changing tobacco habits in the birth cohorts. However, for other sites, viz., hypopharynx, oesophagus and lung, more detailed information on relevant tobacco habits in the birth cohorts is necessary for interpreting the absence of a consistent trend in successive birth cohorts. The recent trends in per capita consumption by type of tobacco (viz., chewing/bidi/cigarette) suggest an emerging cancer pattern in the country at variance with the pattern expected from the current cancer trends in Bombay. Consequently, it is desirable to direct primary cancer prevention programmes especially to cigarette smokers in urban centres and to both bidi and cigarette smokers in the rest of the country.


Br. J. Cancer (1987) 56, 847–852 © The Macmillan Press Ltd., 1987

### Cancers of the upper alimentary and respiratory tracts in Bombay, India: A study of incidence over two decades

K. Jayant[1] & B.B. Yeole[2]

[1]*Cancer Research Institute, Tata Memorial Centre, Parel, Bombay 400012 and* [2]*Bombay Cancer Registry, Indian Cancer Society, Parel, Bombay 400012, India.*

**Summary** Cancers of tongue, oropharynx and larynx in males have registered a decline in incidence over the last two decades in Bombay. This decline has been shown to be a cohort effect. A synoptic measure of risk in each birth cohort, obtained by estimating site-specific cumulative incidence rate over an appropriate age range, was found useful in assessing the risk differential in successive birth cohorts.

The changing pattern in incidence of cancers at several sites *viz.*, tongue, oropharynx, and larynx, where bidi smoking is the predominant risk factor, were in conformity with the pattern expected on the basis of changing tobacco habits in the birth cohorts. However, for other sites, *viz.*, hypopharynx, oesophagus and lung, more detailed information on relevant tobacco habits in the birth cohorts is necessary for interpreting the absence of a consistent trend in successive birth cohorts.

The recent trends in *per capita* consumption by type of tobacco (*viz.*, chewing /bidi/cigarette) suggest an emerging cancer pattern in the country at variance with the pattern expected from the current cancer trends in Bombay. Consequently, it is desirable to direct primary cancer prevention programmes especially to cigarette smokers in urban centres and to both bidi and cigarette smokers in the rest of the country.

Cancers of the upper alimentary and respiratory tracts constitute almost 50% of all cancers in males in Bombay. These cancers are known to be aetiologically associated with the habit of chewing and smoking tobacco. It is relevant to examine the available data for time trends in incidence of these cancers and to attempt an explanation in the light of the changing pattern of tobacco habits in the population.

### Subjects and methods

The Bombay Cancer Registry which was set up in 1964, provides data on chronological trends in incidence of various cancers in the city over a 20 year period (Table I). With a little care in adjusting for differences in classification in the 7th to the 9th revision, of International Classification of Diseases (ICD), the age adjusted rates for various systemic groups could be compared. For specific sites, trends in incidence rates could also be studied in some detail. Furthermore, if the average annual incidences reported for the various periods 1964–66, 1968–72, 1973–77, 1978–82 are considered to represent the incidence at the respective mid points of the given periods, we would have age-specific incidence rates for successive fifth years, *viz.*, 1965, 1970, 1975 and 1980 enabling us to compare the age-specific incidence curves for successive 5 year birth cohorts. For instance, the age-specific rates for 30–34 years in 1964–66, 35–39 in 1968–72, 40–44 years in 1973–77, 45–49 years in 1978–82 could be taken to represent the age-specific incidence rates for 32.5, 37.5, 42.5, and 47.5 years respectively, for those born in 1933, for the purposes of drawing the corresponding age-specific curve.

Although the age-specific incidence curves for cohorts indicate the differences between cohorts, the risk differentials could be better appreciated by having a synoptic measure for the experience of each cohort over the available age range. In the present study, age-specific incidence rates are available for two overlapping 15 year intervals for each cohort and any two successive 5 year birth cohorts have one such interval common to them. Table II shows the possible 5 year age intervals for the various birth cohorts within the time frame for which the incidence data are available and illustrates (by italicising the intervals), how two identical 15

**Table I** Site specific age-adjusted cancer incidence rates for males in Bombay 1964–82

| *Site of malignant neoplasm* | *Age-adjusted incidence rate (world) per $10^5$ per year* | | | |
|---|---|---|---|---|
| | *1964–66*[a] | *1968–72*[b] | *1973–77* | *1978–82* |
| All sites | 139.5 | 143.1 | 142.1 | 147.4 |
| Oral cavity | 21.0 | 19.9 | 16.9 | 17.2 |
| Tongue | 14.0 | 12.6 | 10.2 | 9.7 |
| Mouth (all other parts) | 7.0 | 7.3 | 6.7 | 7.5 |
| Pharynx | 16.6 | 15.8 | 16.4 | 17.7 |
| Oropharynx | 6.1[c] | 5.6 | 4.5 | 3.5 |
| Hypopharynx | 7.3 | 7.7 | 8.7 | 10.0 |
| Digestive organs | 34.8 | 37.8 | 38.6 | 41.4 |
| Oesophagus | 13.0 | 15.2 | 14.7 | 15.0 |
| Respiratory organs | 28.6 | 28.6 | 28.6 | 27.6 |
| Larynx | 13.8 | 13.6 | 12.4 | 10.1 |
| Lung | 13.3 | 13.5 | 14.7 | 15.8 |
| Bone, soft tissue, breast | 3.2 | 4.5 | 5.5 | 4.4 |
| Genito urinary organs | 13.2 | 14.7 | 14.9 | 17.8 |
| Lymphosarcoma and leukaemia | 7.3 | 7.2 | 9.5 | 9.8 |
| All other | 14.7 | 14.5 | 11.7 | 11.3 |

[a]Jussawalla (1970); [b]Waterhouse *et al.* (1976); [c]Refers to ICD. 7,145: tonsils and oral mesopharynx.

**Table II** The age intervals for which incidence data are available for various birth cohorts[a]

| *Birth year of cohort* | *Period of diagnosis* | | | |
|---|---|---|---|---|
| | *1964–66* | *1968–72* | *1973–77* | *1978–82* |
| 1943 | | 25–29 | 30–34 | 35–39 |
| *1938* | 25–29 | *30–34* | *35–39* | *40–44* |
| *1933* | *30–34* | *35–39* | *40–44* | 45–49 |
| 1928 | 35–59 | 40–44 | 45–49 | 50–54 |
| 1923 | 40–44 | 45–49 | 50–54 | 55–59 |
| 1918 | 45–49 | 50–54 | 55–59 | 60–64 |
| 1913 | 50–54 | 55–59 | 60–64 | |

[a]Identical age-intervals are illustrated for one pair of adjacent birth-cohorts by italicising the intervals.

Correspondence: K. Jayant.
Received 8 January 1987; and in revised form, 6 August 1987.

year age intervals are created for two adjacent cohorts. Estimates of cumulative rates (Day, 1976) over 15 year age intervals in steps of 5 years, provide a synoptic measure of risk for each cohort for comparison with the following 5 year older cohort. As usual, here, we consider that the age-specific incidence at the mid point of each 5 year interval is true for the entire 5 year interval for estimating the 15 year cumulative risk per cent.

### Results

The overall age-adjusted incidence rate per 100,000 for cancer at all sites in males was found to remain largely stable over the 19 year period. When the sites were considered by systemic groups it was observed that the incidence of cancer of the oral cavity showed a decline over the years whereas cancers of the pharynx and respiratory organs did not show any appreciable difference. On the other hand, cancers of the digestive and other organs showed some increase. Furthermore, on examining the site specific rates, it was noted that the downward trend in cancers of the oral cavity could be almost entirely attributed to the decline in the incidence of tongue cancer. In pharyngeal cancers, cancers of the oropharynx and hypopharynx seemed to be having opposite trends, the former declining gradually and the latter showing a tendency to increase. In digestive cancers, incidence of cancer of the oesophagus has shown some increase. In respiratory cancers, cancer of the larynx has declined and cancer of the lung has increased.

For the sites which have shown some change in incidence over the years, the age-specific incidence curves for the various birth cohorts from 1943 to 1913 are shown in Figure 1a, b. It can be noticed that for cancers of tongue,

**Figure 1** (a) Age-specific incidence of cancer of the tongue, oropharynx and hypopharynx in cohorts born between 1913 and 1943; (b) Age-specific incidence of cancer of the oesophagus, larynx and lung in cohorts between 1913 and 1943.

oropharynx and larynx, in general, each successive cohort born 5 years later has a lower age-specific incidence within the range of the available data, though there are a few exceptions, as in the case of birth cohorts 1923 and 1918 which have similar rates for tongue cancer in the age group 45–49 years. The differences between birth cohorts would become more obvious, if we were to consider birth cohorts at 10 yearly intervals, one set beginning with 1943 and shown in continuous lines and the other beginning with 1938, shown in broken lines. For cancers of hypopharynx and oesophagus the separation between cohorts is not marked (though a cohort effect is discernible in cohorts born after 1928). In contrast to the above sites, cancer of the lung shows hardly any cohort effect.

Cumulative incidence rates for 15 year intervals are shown for the various birth cohorts for the sites under consideration in Figure 2a, b. (For each pair of cohorts which has a common 15 year interval, the interval for the younger cohort is represented by a continuous line and that for the older cohort by a broken line.) For tongue cancer, the cumulative incidence rate expressed as a precentage, for the 15 year age interval 35–49 years is 0.123 for the 1933 birth cohort, (interval shown as a continuous line) and 0.138 for the 1928 (i.e. 5 year older) birth cohort (interval shown as a broken line); and for the age range 40–54 years it is 0.214 for the 1928 cohort and 0.264 for the 1923 birth cohort. For cancers of the tongue, oropharynx and larynx, for each pair of cohorts having a common interval, the younger cohort has a lower cumulative incidence rate for the specified age range compared to the corresponding cumulative incidence rate for the 5 year older cohort. The percentage reductions in cumulative incidence in successive cohorts compared to 5 year older cohort (over comparable age ranges) are shown in Table III and can be seen to be

**Figure 2** (a) Cumulative incidence rates over two overlapping 15 year-age-ranges for cancers of the tongue, oropharynx and hypopharynx for the various birth cohorts; (b) Cumulative incidence rates over two overlapping 15 year-age-ranges for cancers of the oesophagus, larynx and lung for the various birth cohorts.

L

**Table III** Percentage change in cumulative incidence rates in succesive birth cohorts (1943 to 1918) compared to the corresponding 5 year older cohort for selected cancer sites by age interval

| Birth cohort | Age interval (years) | Sites of cancer | | | | | |
|---|---|---|---|---|---|---|---|
| | | Tongue | Oropharynx | Hypopharynx | Oesophagus | Larynx | Lung |
| 1943 | 25–39 | −32.3 | 0 | −3.2 | −23.5 | −31.2 | −22.9 |
| 1938 | 30–44 | −20.8 | −10.9 | −14.1 | −25.4 | −22.5 | −14.8 |
| 1933 | 35–49 | −10.9 | −24.8 | −13.8 | −13.9 | −29.2 | −7.9 |
| 1928 | 40–54 | −19.1 | −31.1 | −10.6 | −13.7 | −22.5 | +1.5 |
| 1923 | 45–59 | −20.1 | −14.3 | +14.7 | +1.4 | −24.6 | +7.6 |
| 1918 | 50–64 | −7.2 | −20.9 | +19.8 | 0 | −8.5 | +7.4 |

generally appreciable. For cancers of hypopharynx and oesophagus, cumulative incidence is lower for younger cohorts compared to 5 year older cohorts for those born after 1923, whereas for those born before 1923 there is hardly any change or the trend is reversed (shown as *R* in Figure 2a, b). In the case of lung cancer too, the pattern is not consistent. A directional change is seen for cohorts born before 1928 compared to those born after, although the magnitude is small.

### Discussion

Before attempting an interpretation of the trends, it is of importance to evaluate the reliability of the incidence data. Various indices of reliability have been proposed for the purpose *viz.*, proportion with microscopic verification of diagnosis (MV), proportion registered by death certificates (DC) and percentage of deaths in period (DIP) (Waterhouse *et al.*, 1982). For the Bombay Cancer Registry, the percentage of cases with microscopic confirmation for males was 67.8 in 1964–66, and about 67% in the subsequent years. The percentage of cases diagnosed by death certificate alone was 15% when the Registry was set up (1964–66), 14% in 1973–75 and dropped to 9% in the subsequent years as in any continuing Registry. Furthermore, the deaths in period (DIP) to total morbidity cases was 56% in 1973–75 (Waterhouse *et al.*, 1982) and 52% in 1983 and 1984 (National Cancer Registry, 1984). It may be mentioned here that the death registration system in Bombay is supposed to be the best in India with registration of cause of death being complete in 97.1% of cases (Gupta & Rama Roa, 1973). The various indices of reliability for UK (England & Wales, Mersey Region 1975–77) were 53% (MV), 11% (DC), 71% (DIP) and for Connecticut 1973–77, 91% (MV), 1% (DC) and 55% (DIP) (Waterhouse *et al.*, 1982). It appears that cancer registration in Bombay is of an acceptable standard and an interpretation of the observed trends could be attempted.

Several studies in India have shown that chewing and/or smoking tobacco are the main risk factors for cancers of the upper aerodigestive tract. The changing pattern in these cancers could be viewed in the light of prevalent tobacco habits in the various cohorts and the risk ratios associated with specific tobacco habits. Risk ratios in smokers and chewers for several cancer sites have been estimated in two studies (Jussawalla & Deshpande, 1971; Sanghvi, 1981). However, cancer of the base of the tongue which is reported to have characteristics similar to that of oropharyngeal cancer (Paymaster, 1957) is generally grouped with cancer of the oropharynx, in contrast to the international classification which groups the entire tongue under oral cavity. As a result, estimate of risk ratio is available for cancer of the oropharynx inclusive of the base of the tongue and will be considered appropriate for cancers of both oropharynx and tongue (as in Bombay, cancer of the tongue comprises mostly (75%) of cancers at base of the tongue).

It has been shown in the above mentioned studies that the risk ratios in bidi smokers are higher for cancers of the oropharynx (RR = 10.4) and larynx (RR = 7.7) than in chewers (RR = 3.3 and 7.8 for oropharynx and 4.6 for larynx) whereas risk in chewers is higher for cancers of the oral cavity (RR = 6.0 and 3.9) and hypopharynx (RR = 6.2 and 4.5) than in bidi smokers (RR = 2.1 for oral cavity and RR = 2.4 for hypopharynx). For cancer of the oesophagus the risk ratio in both smokers and chewers is similar (~2 fold). Those combining the habit of smoking and chewing have a much higher risk – almost multiplicative compared to those indulging in only the single habit. The risk of the combined habit is particularly high for cancers of the oropharynx (31.7), hypopharynx (16.9), and larynx (20.1).

We could consider some of the Western studies for estimates of risk of cancer at the above sites, specifically, in cigarette smokers. For cancer of the oral cavity, risk estimates are 1.5 (Rothman & Keller, 1972) and 3 (Wynder, 1975). The pharynx is generally considered in conjunction with other sites. One study which groups together the oropharynx and hypopharynx did not show any significant risk for cigarette smokers (Elwood *et al.*, 1984). For cancer of the larynx, the risk ratio was 3 for those smoking 1–15 cigarettes and 6 for those smoking 16–30 (Wynder *et al.*, 1976). It is worthwhile noting that bidi smokers have a much higher risk of oropharyngeal and laryngeal cancers than cigarette smokers.

For lung cancer, both bidi and cigarette smoking are proven risk factors (Notani *et al.*, 1977; Jussawalla & Jain, 1979).

Data on tobacco usage in the general population are not available to study the changes, if any, in the habit pattern and their effect on cancer risk in the different birth cohorts. However, an attempt is made to explain the observed pattern in the light of prevalent tobacco habits in blue collar workers from a cohort study which was carried out in the city between 1971 and 1976 to assess the health consequences of smoking (Jayant *et al.*, 1983). The data on habits of blue collar workers are shown in Table IV, for the age range 35 to 54 years along with the estimated year of birth. By approximating the habits in the different age groups to the habit pattern in appropriate birth cohorts we have limited data on the habits of cohorts born between 1921 and 1936 to interpret the cancer experience of cohorts born between 1913 and 1943. Needless to say the interpretation has to be viewed at a very gross level.

The habit pattern in the various birth cohorts shows that there is a marked decrease in the proportion of both bidi smokers (from 21% to 11.8%) and those with the dual habit of bidi smoking and tobacco chewing ( from 9.4% to 4.2%) in the successively younger cohorts born between 1921 and 1936. Corresponding proportions of cigarette smokers and cigarette smokers plus tobacco chewers show some increase. As seen earlier, bidi smokers with or without the additional habit of tobacco chewing have a much higher risk of cancer of the oropharynx including base of the tongue and larynx compared to cigarette smokers. Consequently, one could

**Table IV** Habit profile in various birth cohorts in blue collar workers

| Habit | Chewing | Smoking | *Age group (in years) and birth year of cohort* 35–39 1936 (n=2,004) % | 40–44 1931 (n=1,577) % | 45–49 1926 (n=1,184) % | 50–54 1921 (n=899) % |
|---|---|---|---|---|---|---|
| None[a] | — | — | 36.3 | 34.3 | 27.9 | 28.0 |
| Single | — | Bidi | 11.8 | 15.2 | 19.5 | 21.0 |
| | — | Cigarette | 12.2 | 12.0 | 9.7 | 9.0 |
| | Tobacco | — | 30.2 | 26.4 | 29.8 | 28.6 |
| | Other | — | 1.5 | 1.5 | 2.2 | 1.3 |
| Dual | Tobacco | Bidi | 4.2 | 7.5 | 7.6 | 9.4 |
| | Tobacco | Cigarette | 2.7 | 1.8 | 2.0 | 2.0 |
| | Other | Bidi | 0.7 | 0.6 | 0.9 | 0.4 |
| | Other | Cigarette | 0.4 | 0.5 | 0.3 | 0.1 |
| All tobacco chewers | | | 37.1 | 35.7 | 39.4 | 40.0 |
| All smokers | | | 32.0 | 37.6 | 40.0 | 40.0 |

[a]Includes ex-smokers and ex-chewers.

**Table V** *Per capita* consumption of raw tobacco in India (g)[a]

| *Tobacco type* | *1951–52* | *1960–61* | *1970–71* | *1980–81* |
|---|---|---|---|---|
| Bidi | 145 | 168 | 155 | 191 |
| Cigarette | 55 | 97 | 133 | 115 |
| Chewing | 140 | 143 | 94 | 54 |
| Total | 556 | 566 | 474 | 541 |

[a]Estimated from data published in Indian Tobacco Statistics, 1975, *Tobacco in India, A Handbook of Statistics* (1983).

expect the corresponding cumulative incidence rates in the successively younger cohorts to show a decline for these cancers. In fact, the observed findings for these cancers are in conformity with the expected trend. On the other hand, as decrease in bidi smoking in the younger cohorts is only partially compensated by increase in cigarette smoking, one would have expected cumulative incidence rates of lung cancer (for which both bidi and cigarette smoking are risk factors) to also show a decrease in successively younger cohorts. However, such a decline is observed only for cohorts born after 1928. Those born before seem to show an opposite trend. There is a need for detailed data on frequency, duration etc. of bidi and cigarette smoking in the birth cohorts in the general population to explain the situation in lung cancer, which does not as yet show a consistent pattern in the successive birth cohorts.

Furthermore, the proportion of total chewers has shown only minimal changes in successive birth cohorts. This could be the reason for the generally stable rates observed in the last two decades for cancers of the oral cavity (excluding the tongue) which is strongly associated with chewing. However, hypopharyngeal cancer which is also chewing dependent has shown some decline in the young cohorts possibly due to the decrease in the dual habit group which has a much higher risk of hypopharyngeal cancer than oral cancer. For cancer of the oesophagus, the attributable risk for chewing and/or smoking is only 50% and therefore the changing pattern of tobacco habits would perhaps not have as marked an effect as on cancer at the other sites under consideration, where the attributable risk is as high as 70 to 84% (Jayant *et al.*, 1977). Even so, the change in cumulative incidence rate in successive cohorts is similar to the pattern observed for hypopharyngeal cancer. The hypothesis, as yet unconfirmed, that oesophageal and hypopharyngeal cancers are associated with chewing quid without tobacco (Jussawalla & Deshpande, 1971) needs to be explored.

In summary, the clear trend in cancers of the tongue, oropharynx and larynx where bidi smoking is the dominant risk factor could be explained on the basis of available data. However, for other sites (excepting the oral cavity), where chewing or cigarette smoking is an equally or more important risk factor than bidi smoking, further data on detailed habit pattern in birth cohorts in the general population are required to elucidate the observed lack of consistency in the trends.

*Future cancer pattern in the country*

As 76% of India's population reside in rural areas, the cancer pattern in the country would depend largely on the cancer trends in rural areas. However, as yet there are no ongoing rural cancer registries. In the absence of relevant data, an attempt may be made to predict the emerging cancer pattern in the country on the basis of tobacco consumption in the population, as the above analysis of the Bombay Registry data lends confirmatory credence to this approach.

The Ministry of Agriculture publishes data (Indian tobacco statistics 1975, 1983) on various types of tobacco annually cleared for domestic consumption (and reflects the actual consumption in the population in view of the stringent import regulations). *Per capita* consumption by type of tobacco were estimated for the period 1951 to 1981 on the basis of decennial census of the population and are shown in Table V. These data could be used to predict the emerging cancer pattern in the country.

It can be observed that the *per capita* consumption of chewing tobacco has steadily decreased over the years and that of bidis has remained steady in the fifties and sixties and increased in the eighties. However, cigarette consumption has steadily increased up to the 1980s. It is also estimated (from the same set of data) that the average number of bidis smoked was around 1,000 per adult per year in the fifties and remained steady up to 1974 and increased to 1,500 in 1976, whereas the average number of cigarettes smoked was 100 per adult per year in the fifties and 190 in the seventies (Sanghvi, 1981). (It would be pertinent to mention here that the national figures for tobacco consumption, particularly of bidi and chewing tobacco, would largely reflect the rural situation). Due to this changing pattern in the consumption of tobacco at national level, one can expect in the future a decline in oral cancer excluding the tongue and an increase in oropharyngeal, tongue, laryngeal and lung cancers in the country. However, in Bombay the available data suggest smokers prefer cigarettes to bidis and there is a decline in bidi smokers. This situation has already resulted in a reduction in cancers of the tongue, oropharynx and larynx (though not lung cancer) in the city. Lung cancer has shown a 16% increase in the 1980s compared to the 1960s. Although for this cancer the trend in birth cohorts is not yet clear, it is likely that lung cancer will overtake all the other tobacco dependent cancers in the city in the absence of primary prevention programmes. In view of the differences in the likely cancer patterns emerging in the city and the country (which is mainly rural), it is desirable to direct primary prevention programmes especially to cigarette smokers in urban centres like Bombay and to both bidi and cigarette smokers in the rest of the country.

### References

DAY, N.E. (1976). A new measure of age standardised incidence rate. In *Cancer Incidence in Five Continents*, Waterhouse, J. *et al.* (eds) p. 443, Scientific Publication no. 15. IARC: Lyon.

ELWOOD, J.M., PEARSON, J.C.G., SKIPPEN, D.H. & JACKSON, S.M. (1984). Alcohol, smoking, social and occupational factors in the aetiology of cancer of the oral cavity, pharynx and larynx. *Int. J. Cancer*, **34**, 603.

GUPTA, R.B. & RAMA RAO, G. (1973). Effect of elimination of different causes of death on expectation of life – Bombay 1960–61. *Indian J. Med. Res.*, **61**, 950.

INDIAN TOBACCO STATISTICS (1975). Directorate of Tobacco Development, Ministry of Agriculture and Irrigation, Government of India.

JAYANT, K., BALAKRISHNAN, V., SANGHVI, L.D. & JUSSAWALLA, D.J. (1977). Quantification of the role of smoking and chewing tobacco in oral, pharyngeal and oesophageal cancers. *Br. J. Cancer*, **35**, 232.

JAYANT, K., NOTANI, P.N. & SANGHVI, L.D. (1983). Health consequences of smoking in two socioeconomic classes in Bombay. *Proc. Fifth World Conf. on Smoking and Health*, Winnipeg.

JUSSAWALLA, D.J. (1970). *Cancer Incidence in Greater Bombay (1964–66)*. The Indian Cancer Society: Bombay.

JUSSAWALLA, D.J. & DESHPANDE, V.A. (1971). Evaluation of cancer risk in tobacco chewers and smokers: An epidemiologic assessment. *Cancer*, **28**, 244.

JUSSAWALLA, D.J. & JAIN, D.K. (1979). Lung cancer in Greater Bombay: Correlation with religion and smoking habits. *Br. J. Cancer*, **40**, 437.

NATIONAL CANCER REGISTRY (1984). Annual report: A project of the Indian Council of Medical Research, New Delhi.

NOTANI, P.N., NAGARAJ RAO, D., SIRSAT, M.V. & SANGHVI, L.D. (1977). A study of lung cancer in relation to bidi smoking in different religious communities in Bombay. *Ind. J. Cancer*, **14**, 115.

PAYMASTER, J.C. (1957). The importance of a uniform clinical classification and staging of carcinomas of the upper digestive tract. *Indian J. Surg.*, **19**, 1.

ROTHMAN, K.J. & KELLER, A. (1972). The effect of joint exposure to alcohol and tobacco on risk of cancer of the mouth and pharynx. *J. Chron. Dis.*, **25**, 711.

SANGHVI, L.D. (1981). Cancer epidemiology: The Indian scene. *J. Cancer Res. Clin. Oncol.*, **99**, 1.

TOBACCO IN INDIA, A HANDBOOK OF STATISTICS (1983). Directorate of Tobacco Development, Ministry of Agriculture and Irrigation, Government of India.

WATERHOUSE, J., MUIR, C., CORREA, P. & POWELL, J. (eds) (1976). *Cancer Incidence in Five Continents*, Scientific publication no. 15, IARC: Lyon.

WATERHOUSE, J., MUIR, C., SHANMUKHARATNAM, K. & POWELL, J. (eds) (1982). *Cancer Incidence in Five Continents*, Vol. **IV**, Scientific publication no. 42, IARC: Lyon.

WYNDER, E.L., BROSS, I.J. & FELDMAN, R.M. (1975). A study of the etiologic factors in cancers of the mouth. *Cancer*, **10**, 1300.

WYNDER, E.L., COVEY, L.S., MABUCHI, K. & MUSHINSKI, M. (1976). Environmental factors in cancers of the larynx: A second look. *Cancer*, **38**, 1591.